# The Gut Microbiome in Human Obesity: A Comprehensive Review

**DOI:** 10.3390/biomedicines13092173

**Published:** 2025-09-05

**Authors:** Alejandro Borrego-Ruiz, Juan J. Borrego

**Affiliations:** 1 Departamento de Psicología Social y de las Organizaciones, Universidad Nacional de Educación a Distancia (UNED), 28040 Madrid, Spain; 2 Departamento de Microbiología, Universidad de Málaga, 29071 Málaga, Spain; jjborrego@uma.es

**Keywords:** gut microbiome, obesity, microbial metabolites, dietary influence, precision nutrition, microbiome-based therapeutics, novel treatments

## Abstract

An estimated 2.6 billion individuals are currently living with overweight or obesity, and this number is projected to exceed 4 billion by 2035. Consequently, unless this increasing trajectory is effectively addressed, the trend is expected to continue in the coming years. The gut microbiome has emerged as a central regulator of host metabolism and energy homeostasis, making its detailed characterization crucial for the advancement of innovative therapeutic strategies and for elucidating mechanisms underlying metabolic health and disease. This review examines human obesity through the lens of the gut microbiome, providing a comprehensive overview of its role by addressing gut microbiome alterations, microbiome-driven mechanisms, dietary influences, prebiotic effects, microbiome-based therapeutics, and other approaches in the treatment of obesity and related metabolic disorders. The composition of the gut microbiome is altered in obesity and characterized by reduced microbial diversity and inconsistent shifts in dominant bacterial phyla, which collectively contribute to metabolic dysregulation. The gut microbiome influences obesity through multiple mechanisms. These include regulating energy balance and insulin sensitivity via short-chain fatty acids, inducing chronic inflammation, modulating metabolic and appetite genes, altering bile acid signaling, and promoting fat storage by inhibiting fasting-induced adipose factor. Dietary patterns exert a profound influence on gut microbiome composition and function, with plant-based diets conferring protective effects against obesity and its comorbidities. Microbiome-based therapeutics, including probiotics, synbiotics, and fecal microbiota transplantation, have demonstrated potential in modulating key metabolic and inflammatory pathways associated with obesity. As the scientific understanding of the human gut microbiome continues to advance, the integration of microbiome-based therapies into standard clinical practice is poised to become increasingly feasible and therapeutically transformative, particularly for obesity, a complex condition that demands innovative and customized interventions.

## 1. Introduction

Obesity results from the chronic accumulation of adipose tissue, primarily driven by a sustained imbalance between caloric intake and energy expenditure [1]. At present, an estimated 2.6 billion individuals, equivalent to 40% of the global population, are living with overweight or obesity. It is projected that this number could exceed 4 billion by 2035, approaching half of the world’s population [2,3]. The persistent rise in obesity rates reflects the convergence of numerous interrelated factors, including but not limited to high-fat diets, sedentary lifestyles, neurohormonal dysregulation, and genetic and epigenetic susceptibility, as well as environmental and behavioral influences. Unless this increasing trajectory is effectively addressed, the trend is expected to continue in the coming years [4].

Individuals with obesity are predisposed to a multitude of obesity-related pathologies, including metabolic dysfunction-associated steatotic liver disease (formerly non-alcoholic fatty liver disease), metabolic syndrome (e.g., dyslipidemia, insulin resistance, hypertension, and elevated fasting glucose), type 2 diabetes mellitus (T2D), hepatic disorders, cardiovascular diseases, and certain cancers [5,6,7]. Moreover, obesity has been associated with diminished life satisfaction and the emergence of adverse mental health conditions, such as anxiety and depression [8]. Consequently, obesity constitutes a major contributor to the increased morbidity and mortality rates observed in contemporary populations [9]. This can be explained by evidence indicating that excessive adipose tissue accumulation in obesity activates cells of the innate immune system, which in turn promotes a state of chronic low-grade inflammation [10].

Traditionally linked to caloric intake and physical inactivity, obesity is now increasingly recognized as being influenced by the gut microbiome (GM), a diverse and dynamic microbial community residing in the gastrointestinal tract [10,11]. Emerging evidence indicates that the GM affects host metabolism through bioactive metabolites, which may regulate pivotal processes such as insulin sensitivity, lipogenesis, neurohormonal signaling, and systemic inflammation [5,12]. However, although research links the GM to obesity, an integrative synthesis connecting microbial mechanisms, dietary interventions, and GM-targeted therapies remains lacking. In this context, the prevailing hypothesis suggests that an imbalance in GM composition, known as dysbiosis, contributes to the activation of numerous signals via multiple pathways, resulting in obesity and its related complications. Indeed, dysbiosis plays a crucial role by influencing hunger regulation, satiety, and nutrient absorption [13]. It also promotes oxidative stress, inflammatory responses, increased adiposity, and metabolic dysfunction, including disturbances in glucose metabolism and adipocyte distribution. Despite these insights, the development of effective screening, diagnostic, and therapeutic approaches for obesity and its comorbidities remains limited, largely due to incomplete understanding of its underlying pathophysiology [5].

Classical approaches to obesity management have included pharmacotherapy, bariatric surgery, and lifestyle interventions [14]. Although significant progress has been made in pharmacological treatments in recent years [15,16], most anti-obesity drugs remain contraindicated for use during adolescence and pregnancy [17]. In turn, bariatric surgery carries considerable risks and potential complications [18]. Evidence-based lifestyle approaches typically encompass a combination of dietary modification, increased physical activity, and behavioral change therapies, which synergistically support sustainable weight management and contribute to improved metabolic outcomes [19,20,21]. Despite lifestyle-based interventions increasing the likelihood of achieving clinically significant weight loss in individuals with obesity, long-term weight maintenance remains a major obstacle, as weight regain over time is common, even among individuals who adhere to treatment protocols [22]. In light of these challenges, emerging therapeutic approaches, such as probiotic and prebiotic supplementation, brown adipose tissue transplantation, fecal microbiota transplantation (FMT), glucagon-like peptide-1 receptor agonists, poly-agonist pharmacotherapies, ultrasound stimulation of the vagus nerve, and precision nutrition strategies, have shown promise for clinical application [15,23,24,25,26,27,28].

Excessive adiposity significantly impairs quality of life and is associated with numerous comorbidities, collectively heightening the risk of preventable mortality. The GM has emerged as a central regulator of host metabolism and energy homeostasis, making its detailed characterization crucial for the advancement of innovative therapeutic strategies and for elucidating mechanisms underlying metabolic health and disease. Understanding these microbiome-mediated mechanisms is therefore essential for developing targeted interventions to manage obesity. In fact, these insights may also reveal that modulation of the GM can offer broader human health outcomes beyond metabolism. Based on these considerations, it is hypothesized that GM dysbiosis contributes causally to obesity by modulating energy balance, inflammation, and metabolic signaling, highlighting a knowledge gap in current obesity research. This review offers a novel and integrative perspective by connecting current evidence on GM functions with potential GM-focused strategies for obesity therapy. More specifically, it examines human obesity through the lens of the GM, providing a comprehensive overview of its role by addressing GM alterations, microbiome-driven mechanisms, dietary influences, prebiotic effects, microbiome-based therapeutics, and other approaches in the treatment of obesity and related metabolic disorders.

## 2. Gut Microbiome Composition in Individuals with Obesity

The composition and function of the human GM play a pivotal role in the development and management of obesity. Several studies have demonstrated that GM composition differs not only between obese and lean subjects but also across geographic regions, with marked variations observed between countries [29]. A review of the literature highlights consistent alterations in the GM of individuals with obesity compared to eutrophic subjects, notably a decrease in microbial richness and diversity [30,31,32]. These compositional changes have been linked to increased adiposity, dyslipidemia, heightened low-grade inflammation, and impaired glucose metabolism, underscoring the relevance of GM alterations in obesity pathophysiology [33].

In terms of GM composition, individuals with obesity often exhibit an increased *Bacillota*/*Bacteroidota* (B/B) ratio in their fecal microbiota, regardless of dietary intake [34,35]. However, this microbial signature is not consistently observed, likely due to confounding factors that influence GM composition, including fasting status, dietary pattern, antibiotic use, age, geographic location, exercise habits, genetic background, and methodological or clinical variables [36,37]. In this context, a decreased B/B ratio was reported in overweight and obese individuals without dietary restrictions [38], suggesting that body mass index (BMI)-related differences in GM composition may not follow a universal pattern [39]. These discrepancies may stem from interpretive bias caused by methodological differences in sample processing and DNA sequencing. Alternatively, they could reflect insufficient characterization of the study participants, especially the omission of lifestyle-related factors known to affect GM composition and diversity [40].

In some cases, differences in GM have been detected at the genus and family levels within the same cohort, without significant variation at the phylum level. For instance, a recent study in Korean adolescents found that individuals with normal weight had higher levels of *Bacteroides*/*Bacteroidaceae*, whereas obese participants exhibited increased levels of *Prevotella*/*Prevotellaceae*. However, the relative abundance of *Bacillota*, *Bacteroidota*, and *Pseudomonadota*, as well as the B/B ratio, did not differ significantly between groups [37]. Although there is a general consensus on the increase in Bacillota [41], other studies suggest that obesity risk may be more closely linked to reductions in *Bifidobacterium* spp. (Actinomycetota) [35] or *Akkermansia muciniphila* (Verrucomicrobiota) [42], rather than changes in the B/B ratio.

Other studies have revealed marked differences in GM bacterial composition among individuals with obesity. Kasai et al. [43] found that certain bacterial species, including *Blautia hydrogenotrophica*, *Blautia* (formerly *Ruminococcus*) *obeum*, *Coprococcus catus*, *Eubacterium ventriosum*, and *Ruminococcus bromii*, were linked to obesity in a Japanese population. In turn, Gao et al. [44] reported that in individuals with obesity, beneficial bacteria such as *Bifidobacterium*, *Faecalibacterium*, and butyrate-producing *Ruminococcaceae* are significantly reduced, whereas potential opportunistic pathogens, including *Escherichia*/*Shigella* and *Fusobacterium*, are increased. In addition, Crovesy et al. [45] conducted a systematic review of 32 studies and reported that individuals with obesity exhibit an increased abundance of members of the phyla Bacillota, Fusobacteriota, and Pseudomonadota, as well as the species *Limosilactobacillus* (formerly *Lactobacillus*) *reuteri*. Conversely, a decline has been observed in the abundance of the phylum Bacteroidota and species such as *A*. *muciniphila*, *Faecalibacterium prausnitzii*, *Methanobrevibacter smithii*, *Lactiplantibacillus* (formerly *Lactobacillus*) *plantarum*, and *Lacticaseibacillus* (formerly *Lactobacillus*) *paracasei*.

In a review conducted by Cani et al. [46], it was reported that members of the genera *Clostridium*, *Lactobacillus*, and *Ruminococcus* are elevated in obese patients, while *F*. *prausnitzii* is more prevalent in healthy individuals and reduced in those with obesity. Similarly, Duan et al. [30] observed significant differences in GM composition between obese patients and control subjects. At the phylum level, Bacillota, Bacteroidota, Actinomycetota, and Fusobacteriota showed significant disparities between the groups. At the genus level, 16 major genera exhibited notable differences, with *Prevotella*, *Megamonas*, *Fusobacterium*, and *Blautia* markedly increased in obese patients. Conversely, the remaining 12 genera, specifically *Faecalibacterium*, *Lachnospiracea_incertae_sedis*, *Clostridium XIVa*, *Coprococcus*, *Gemmiger*, *Ruminococcus*, *Parabacteroides*, *Bifidobacterium*, *Clostridium IV*, *Alistipes*, *Oscillibacter*, and *Barnesiella*, demonstrated reduced prevalence. At the species level, nine species showed significant differences, with an increased abundance of *Megamonas funiformis*, *Segatella* (formerly *Prevotella*) *copri*, and *Fusobacterium mortiferum* in obese subjects, whereas *Bacteroides uniformis*, *F. prausnitzii*, *Fusicatenibacter saccharivorans*, *Barnesiella intestinihominis*, *Parabacteroides distasonis*, and *Alistipes putredinis* were decreased.

Palmas et al. [47] conducted a study characterizing the GM signatures of overweight and obese individuals compared to normal-weight controls in Sardinia, Italy. Their findings revealed a significant reduction in the relative abundance of multiple Bacteroidota taxa within the microbial communities of obese patients, including members of the families *Flavobacteriaceae*, *Porphyromonadaceae*, and *Sphingobacteriaceae*, as well as the genera *Bacteroides*, *Flavobacterium*, *Parabacteroides*, *Pedobacter*, and *Rikenella*. However, several Bacillota taxa exhibited a marked increase in the same subjects, including members of the families *Gemellaceae*, *Lachnospiraceae*, *Paenibacillaceae*, *Streptococcaceae*, and *Thermicanaceae* and the genera *Acidaminococcus*, *Eubacterium*, *Gemella*, *Megamonas*, *Megasphaera*, *Mitsuokella*, *Ruminococcus*, *Streptococcus*, *Thermicanus*, and *Veillonella*. Body fat (BF) percentage and waist circumference (WC) showed a negative correlation with the abundance of Bacteroidota taxa. In contrast, Bacillota taxa showed a positive correlation with BF and a negative correlation with muscle mass and/or physical activity levels. Furthermore, the obese group exhibited an increased relative abundance of several bacterial taxa within the *Enterobacteriaceae* family, known for their endotoxic activity, compared to normal-weight controls.

**Figure 1:**
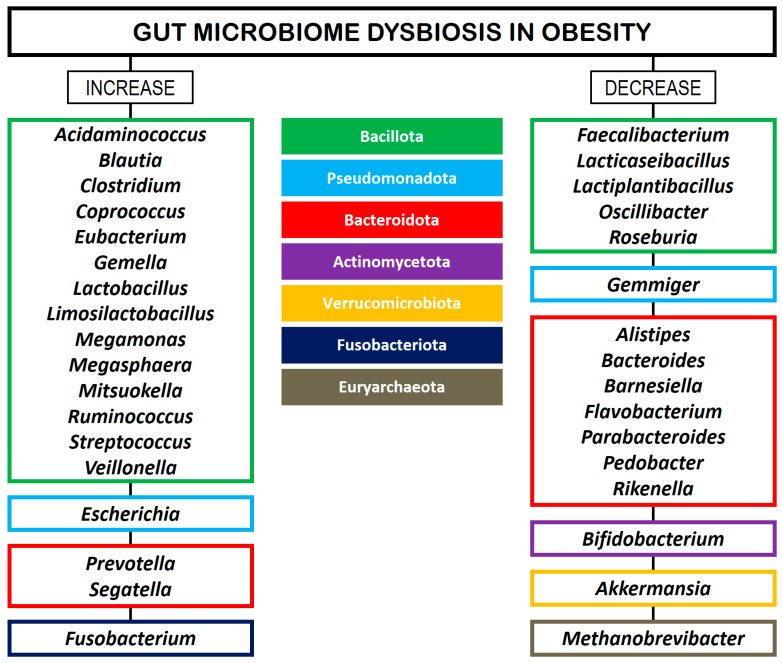
GM dysbiosis in patients with obesity. The left column shows the major bacterial genera that are increased in abundance relative to controls. The right column shows the major bacterial genera that are decreased in abundance relative to controls. The bacterial phyla are color-coded in the middle column.

In a systematic review, Xu et al. [48] identified bacterial taxa specifically associated with obesity and metabolic disorders across Western and Eastern populations. The phylum Pseudomonadota was the most frequently reported in obese individuals, while the genera *Faecalibacterium*, *Akkermansia*, and *Alistipes* were negatively correlated with obesity. In turn, *Ruminococcus* and *Prevotella* were identified as obesity-related genera in studies conducted in Western regions. Conversely, these genera were found to be lean-related in studies conducted in Eastern regions. Moreover, the genera *Roseburia* and *Bifidobacterium* were associated with a lean BMI in the Eastern population, while *Lactobacillus* was associated with obesity in the Western population. Yan et al. [49] employed whole-genome shotgun sequencing to reveal that the GM of patients with high visceral fatty areas (VFAs) exhibited a distinct microbial composition compared to those with low VFAs, characterized by an increased abundance of 18 bacterial species in the high-VFA group and 9 species that were more prevalent in the low-VFA group. A total of 16 gut microbial species exhibited strong correlations with VFA, with *E. coli* showing the highest association, followed by *Bifidobacterium longum*, *Dialister succinatiphilus*, *Eubacterium hallii*, *Escherichia* unclassified, *Mitsuokella* unclassified, *Ruminococcus gnavus*, and *Mediterraneibacter* (formerly *Ruminococcus*) *torques*. Furthermore, only two species (i.e., *E*. *hallii* and *Solobacterium moorei*) were found to be positively linked with BMI, while two species (i.e., *D*. *succinatiphilus* and *Mitsuokella* unclassified) demonstrated a positive correlation with WC. Figure 1 provides a summary of GM alterations in patients with obesity, according to several studies [30,43,44,45,46,47,48].

## 3. Gut Microbiome-Induced Mechanisms in Obesity

Various mechanisms have been proposed to explain the association between GM composition and the pathogenesis of obesity [50]. The first mechanism involves the microbial fermentation of non-digestible carbohydrates into metabolic byproducts, primarily short-chain fatty acids (SCFAs) such as acetate, butyrate, and propionate [51,52]. SCFAs are produced through anaerobic fermentation and function as ligands for G-protein-coupled receptors (GPRs). Acetate binds GPR43, butyrate binds GPR41, and propionate binds both. In states of dysbiosis and obesity, the expression of these receptors may be downregulated, contributing to hepatic lipogenesis and disruptions in energy homeostasis [53,54]. On the other hand, SCFAs have also been shown to promote fat oxidation and energy expenditure while inhibiting lipolysis. These effects support thermogenesis in brown adipose tissue and the browning of white adipose tissue [55], as well as enhanced insulin sensitivity [56]. Moreover, SCFAs have been implicated in the upregulation of peroxisome proliferator-activated receptors (PPARs), which play a key role in adipogenesis regulation [57].

A human pilot study demonstrated that oral butyrate supplementation positively influences glucose metabolism in lean individuals but not in those with metabolic syndrome, likely due to altered SCFA handling in insulin-resistant subjects [58]. Similarly, Li et al. [59] reported that butyrate can suppress appetite and activate brown adipose tissue through the gut–brain neural axis. Additional studies have identified associations between SCFA levels and improved insulin sensitivity, as well as regulatory effects on adipose tissue development, lipid storage, and substrate metabolism in the liver and skeletal muscle [60]. SCFAs that are not absorbed or metabolized by intestinal epithelial cells are transported to the liver via the portal vein, where they serve as substrates for gluconeogenesis, lipogenesis, and cholesterologenesis [61]. In addition, SCFAs have been shown to inhibit histone deacetylase activity, thereby influencing epigenetic modifications such as histone acetylation and methylation [62]. These changes can modulate the expression of genes involved in lipid metabolism and contribute to the restoration of chromatin structure and function [63].

**Figure 2:**
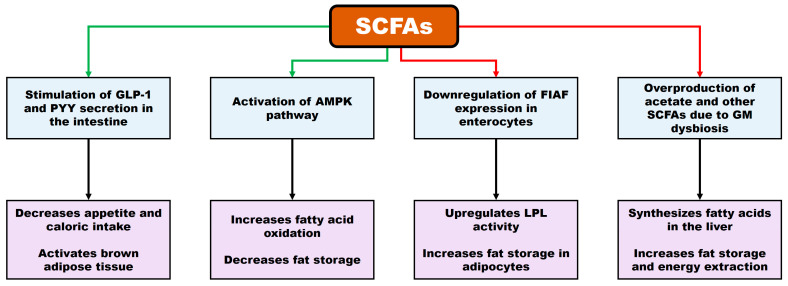
Mechanisms involved in the dual role of SCFAs in obesity. Green arrows: activation processes. (i) SCFAs promote the secretion of hormones such as glucagon-like peptide-1 (GLP-1) and peptide YY (PYY), reducing appetite and, consequently, caloric intake. They also activate brown adipose tissue. (ii) SCFAs stimulate the AMP-activated protein kinase (AMPK) pathway, enhancing fatty acid oxidation and decreasing fat accumulation. Red arrows: inhibition processes. (i) SCFAs downregulate fasting-induced adipose factor (FIAF) expression in enterocytes, increasing lipoprotein lipase (LPL) activity and promoting lipid storage in adipocytes. (ii) Excessive SCFA production induces fatty acid synthesis in the liver, contributing to fat accumulation and increased energy extraction.

According to Iqbal et al. [3], SCFAs exhibit a dual role in obesity, exerting both anti-obesogenic and pro-obesogenic effects. (i) SCFAs promote the secretion of glucagon-like peptide-1 (GLP-1) and peptide YY (PYY), hormones that suppress appetite and reduce caloric intake. (ii) They also activate the AMP-activated protein kinase (AMPK) pathway, enhancing fatty acid oxidation and decreasing fat accumulation. (iii) In contrast, in the context of dysbiosis, SCFAs have been shown to downregulate fasting-induced adipose factor (FIAF) expression in enterocytes, thereby increasing lipoprotein lipase (LPL) activity and promoting lipid storage in adipocytes. (iv) Excessive SCFA production, particularly acetate, can be utilized by the liver for fatty acid synthesis, contributing to lipid accumulation and increased energy extraction. A schematic representation of the dual role of SCFAs in obesity is shown in Figure 2 (modified from Iqbal et al. [3]).

The second mechanism involves the capacity of the GM to modulate circulating lipopolysaccharide (LPS) levels by impairing epithelial barrier integrity, thereby initiating endotoxemia and the onset of moderate chronic systemic inflammation [64,65]. Once in the bloodstream, LPS binds to LPS-binding protein (LBP), which facilitates its recognition by the CD14 receptor. This interaction subsequently activates adipose tissue macrophages through the Toll-like receptor 4 (TLR4) pathway. LPS-TLR4 binding has been shown to upregulate genes involved in immune responses via the nuclear factor-kappa B (NF-κB) signaling pathway, promoting the secretion of pro-inflammatory cytokines such as tumor necrosis factor-alpha (TNF-α), interleukin-1β (IL-1β), and interleukin-6 (IL-6) [66,67]. This persistent low-grade inflammation, termed metabolic inflammation, impairs insulin signaling in peripheral tissues and contributes to systemic insulin resistance, which is a hallmark of obesity and metabolic syndrome [3]. Moreover, these inflammatory processes can drive genetic and epigenetic modifications that elevate the risk of obesity-related comorbidities [63]. In murine models, a single acute injection of LPS has been shown to enhance glucose disposal and glucose-stimulated insulin secretion through activation of the GLP-1 pathway [68].

The third mechanism is based on the hypothesis that the GM can modulate host genes implicated in energy storage and utilization, favoring fat accumulation [69]. In this context, Li et al. [70] conducted a comprehensive metagenomic analysis of 192 patients and reported functional gene variations in *Bacteroides* and *Prevotella* related to amino acid and carbohydrate metabolism. In addition, the authors identified a human genetic variant (rs878394) in the lysophospholipase-like 1 (*LYPLAL1*) gene, which is associated with BF distribution, insulin sensitivity, and the relative abundance of *Prevotella*. The GM has also been shown to regulate appetite and satiety through vagal nerve activation and immune–neuroendocrine signaling pathways [71]. Notably, lower levels of anorexigenic hormones involved in appetite suppression, such as GLP-1 and PYY, have been consistently observed in individuals with obesity [72].

The GM also plays a pivotal role in modulating bile acid (BA) metabolism, thereby influencing liver triglyceride (LT) levels and glucose homeostasis through the activation of the farnesoid X receptor (FXR) [73]. FXR activation in both the liver and intestines has been shown to inhibit hepatic lipogenesis, improve insulin sensitivity, and enhance energy expenditure, ultimately contributing to the mitigation of obesity risk. In addition, BAs have been implicated in preserving intestinal barrier integrity, thereby limiting LPS translocation into the systemic circulation and reducing the onset of chronic low-grade inflammation, which constitutes a key contributor to the pathogenesis of obesity and metabolic syndrome [74].

Furthermore, the GM may inhibit the expression of the *FIAF* gene, thereby increasing LPL activity and promoting lipid accumulation in white adipose tissue [75]. FIAF, which constitutes a protein produced by adipose tissue, the gastrointestinal tract, the liver, and skeletal muscle in response to fasting, plays a pivotal role in lipid metabolism by inhibiting LPL, which reduces triglyceride uptake in adipose and muscle tissues [76]. This inhibitory effect limits fatty acid uptake, potentially preventing excessive fat storage and serving as a mechanism to attenuate obesity [77].

## 4. Impact of Diet on Obesity via the Gut Microbiome

In the context of obesity management, short-term weight loss is primarily driven by calorie restriction rather than diet composition, with the most effective diet being the one that the individual can adhere to [78]. However, beyond caloric intake, dietary quality plays a pivotal role in obesity management and related health outcomes, with patterns such as the Mediterranean diet, vegetarian diets, ketogenic diets, and dairy-inclusive approaches showing benefits in weight control, metabolic health, and inflammation reduction [79,80,81,82,83,84,85,86,87]. Moreover, dietary quality is crucial in obesity-related conditions such as asthma, where high-fat, low-fiber diets are linked to increased airway inflammation and impaired medication responsiveness [20]. The Mediterranean diet, characterized by consumption of high-quality plant-based foods and moderate fat intake, demonstrates weight-loss effects comparable to low-fat and low-carbohydrate diets, while offering added cardiometabolic benefits and long-term sustainability when combined with energy restriction and physical activity [79,80]. Vegetarian diets are also associated with lower BMI and favorable weight outcomes, with key contributors including fiber content, lower caloric density, microbiota regulation, and the release of gastrointestinal appetite-regulating hormones [81,82,83]. Ketogenic diets, via mechanisms such as appetite suppression, reduced lipogenesis, increased lipolysis, and enhanced fat oxidation, have shown short- to medium-term benefits in terms of weight loss and cardiometabolic health in obese patients, although careful monitoring and a gradual transition to a normal diet are essential for safety and adherence [84,85]. In addition, dairy-inclusive, energy-restricted diets may enhance fat loss while preserving lean mass, with growing evidence supporting the benefits of whole-fat and fermented dairy products for body composition and cardiometabolic health [86,87].

Regarding diet outcomes, the GM has become a central focus in recent research due to its substantial contribution to obesity and related comorbidities. GM dysbiosis has been demonstrated to impact adiposity and glucose metabolism, thereby promoting obesity. Furthermore, modulating the GM through dietary strategies has emerged as a promising therapeutic approach in obesity management [5,6]. A high-calorie diet rich in fats and sugars, typical of the Western dietary pattern, combined with a sedentary lifestyle, has been identified as a major risk factor for obesity and its related metabolic disturbances [88]. Such dietary and lifestyle patterns induce the accumulation of adipose tissue, leading to an increase in visceral and subcutaneous fat. Once adipose tissue reaches its storage capacity, excess energy such as triglycerides leads to an increase in blood lipids and ectopic fat accumulation [89]. This progressive adiposity is accompanied by enhanced adipogenesis and the release of pro-inflammatory cytokines and adipokines, which are central to the pathogenesis of obesity-related disorders [90]. In contrast, the Mediterranean diet has been widely studied for its demonstrated efficacy in ameliorating chronic conditions, including T2D and metabolic syndrome [91,92]. These positive effects are largely attributable to the high intake of plant-based foods rich in dietary fiber and antioxidant compounds, such as terpenes and flavonoids, which enhance the production of SCFAs by the GM [93,94]. Consistently, elevated levels of SCFAs have also been observed in individuals following vegetarian diets [95].

High-calorie diets have been shown to modulate GM composition by increasing the abundance of Bacillota members while concurrently decreasing Bacteroidota abundance [96,97]. This shift in the B/B ratio has been particularly noted in obese animal models carrying leptin gene mutations, in contrast to their lean counterparts without such mutations, indicating a link between obesity and altered microbial diversity [98]. Despite the existence of numerous studies documenting variations in the B/B ratio among obese adults, other evidence suggests that obesity is more broadly characterized by an increased prevalence of Actinomycetota and Bacillota phyla, along with a decreased prevalence of species within the Bacteroidota and Verrucomicrobiota phyla, including *A. muciniphila*, as well as a reduction in *F. prausnitzii* [99,100].

Several human studies have shown that a high-fat diet can reduce the abundance of *Bacteroides* spp., *Bifidobacterium* spp., *Clostridium coccoides*, and *Eubacterium rectale* in individuals with obesity. However, these alterations can be reversed via dietary interventions [32,101,102]. Thingholm et al. [103] observed that the abundance of *L. reuteri* in the GM of obese patients was substantially elevated compared to other bacterial genera such as *Akkermansia*, *Alistipes*, *Faecalibacterium*, and *Oscillibacter*, as well as the archaeal species *M*. *smithii*, which tended to decrease. In addition, increased sugar intake has been linked to GM dysbiosis, characterized by a rise in the relative abundance of intestinal Pseudomonadota and a reduction in Bacteroidota, contributing to pro-inflammatory effects and impaired epithelial barrier function [104]. An in vivo study in obese adolescents undergoing a calorie-restricted diet combined with increased physical activity demonstrated that weight-loss interventions induced alterations in GM composition. As observed in corresponding animal studies, these changes, which include increased inflammation and intestinal permeability, resulted in endotoxemia, enhanced fat accumulation, and steatosis [105].

Dietary fiber intake plays a pivotal role in maintaining host health and regulating body weight (BW). Adoption of a vegetarian diet, characterized by a high intake of indigestible fibers, has been shown to decrease the β-diversity of the human GM. This decrease is linked to an increased abundance of the genera *Prevotella* and *Faecalibacterium*, alongside a reduced prevalence of *Bacteroides* [95]. While high *Prevotella* abundances have been demonstrated to promote weight loss in healthy adults with overweight [106], *F*. *prausnitzii* may play a pivotal role in obesity and related metabolic disorders, as it is frequently found in lower abundance in obese subjects and it is associated with reduced inflammation and enhanced gut barrier integrity and has even been shown to reduce appetite [107]. In addition, plant polyphenols increase the populations of *Bifidobacterium* and *Lactobacillus*, which exert anti-inflammatory effects, improve metabolic parameters, and confer cardiovascular protection in humans [108,109]. This modulation of the GM is particularly relevant in the context of obesity, as chronic low-grade inflammation and cardiovascular risk are common comorbidities associated with excessive adiposity [110]. Furthermore, fermented vegetables have been proposed as potential therapeutic agents in managing obesity, as they are particularly rich in *Lactobacillus* species, reduce cholesterol, inhibit adipogenesis, and have anti-diabetic properties [111].

Ketogenic diets are normocaloric, high-fat, and very low-carbohydrate dietary patterns that induce a state of ketosis [112]. This state leads to a reduction in insulin levels due to carbohydrate restriction, thereby suppressing lipogenesis and fat storage, while depleting glucose reserves. Although ketogenic diets are generally normocaloric, they are frequently modified to very-low-calorie versions in certain applications, further enhancing fat loss and other metabolic benefits [108]. The therapeutic effects of ketogenic diets in obese patients are likely mediated by their capacity to augment fatty acid oxidation and lipolysis while suppressing de novo lipogenesis, as well as by modulating hepatic glucose metabolism through decreased circulating insulin and increased glucagon levels, which together inhibit gluconeogenesis and promote elevated resting energy expenditure [112]. Recent studies have emphasized the pivotal involvement of the GM in mediating the effects of the ketogenic diets [113,114]. In human subjects, these dietary patterns have been shown to alter GM composition by decreasing the abundance of the phylum Bacillota and increasing the abundance of members belonging to the phylum Bacteroidota [115,116]. At the genus level, decreases in the abundance of *Bifidobacterium* and *Lachnobacterium*, as well as increases in *Akkermansia* and *Slackia*, have been reported [108,117]. Specifically, *A*. *muciniphila* may play a significant role in obesity and related metabolic disorders, as it can help to prevent weight gain, reducing visceral fat and improving glucose homeostasis [118]. Moreover, Gong et al. [119] noted that *Bifidobacterium* substantially decreased in visceral obesity, with serum uric acid potentially acting as a mediator between decreased *Bifidobacterium* and increased visceral adipose tissue, which suggests that supplementation with this microorganism might be a crucial complement for ketogenic diets in order to promote visceral adipose tissue reduction.

## 5. The Role of Prebiotics in Obesity

Prebiotics are typically polysaccharides defined as substances that induce specific changes in the composition and/or function of the GM, thereby conferring health benefits to the host [120]. To qualify as prebiotics, these substances must meet three criteria: (i) resistance to digestion by host enzymes, gastric acid, and bile; (ii) the ability to selectively stimulate the growth and/or activity of beneficial commensal microbiota; and (iii) fermentability by the GM [121].

Administration of various prebiotics has been shown to alter metabolic functions and GM composition, leading to increased levels of the anorexigenic gastrointestinal peptides GLP-1 and PYY, alongside decreased concentrations of the orexigenic hormone ghrelin [122]. Specifically, prebiotics such as inulin, fructans, and oligofructose promote the proliferation of beneficial bacteria, including lactobacilli and bifidobacteria [123]. Preclinical studies have linked *Bifidobacterium* species to reduced FM, lower BW gain, and diminished inflammation and metabolic endotoxemia [124]. In addition, prebiotic supplementation has been reported to increase the abundance of *A*. *muciniphila*, a species positively associated with improved insulin sensitivity, reduced FM gain, lower systemic inflammation, decreased metabolic endotoxemia, and regulation of energy homeostasis [42,125,126]. These findings in animals have been corroborated by clinical evidence demonstrating that oligofructose intake modulates GLP-1, ghrelin, and PYY levels in humans, thereby reducing hunger sensations and postprandial glucose fluctuations [122,127,128]. In a seminal study, Genta et al. [129] reported that consumption of oligofructose-rich syrup derived from yacon (*Smallanthus sonchifolius*) roots increased satiety and resulted in reductions in BW, BMI, and WC among obese premenopausal women. This intervention also decreased low-density lipoprotein (LDL) cholesterol and fasting insulin levels. Similarly, supplementation with inulin-type fructans in obese women significantly decreased BW, WC, BMI, homeostasis model assessment for insulin resistance (HOMA-IR), and fasting insulin, while improving satiety compared to the placebo group [130]. Table 1 summarizes the effects of various prebiotics administered in both animal models and human studies.

## 6. Microbiome-Based Approaches in Obesity Management

The GM offers a novel perspective on potential therapies by linking inflammation, energy homeostasis, metabolism, and obesity. Several reviews have extensively discussed microbiota modulation strategies [5,148], proposing various microbial approaches to restore gut dysbiosis associated with obesity. Among these, probiotics and synbiotics remain the primary treatment modalities, while FMT has recently shown promising efficacy.

### 6.1. Probiotics and Synbiotics

Probiotics are defined as live microorganisms which, when administered in adequate amounts, confer health benefits to the host [149]. Probiotics have the capacity to modulate the GM, influencing energy and lipid metabolism [150], which can lead to reduced insulin resistance and improved satiety [151]. Several studies have reported that probiotics may aid weight loss by inhibiting adipogenesis and lowering fasting blood glucose levels in obese individuals [109]. In addition, probiotics contribute to cardiovascular health by reducing cholesterol concentrations [152]. They also enhance gut barrier integrity and immunomodulatory functions, while exhibiting antibacterial properties [153]. Furthermore, probiotics can rapidly colonize the gut, promoting lipid metabolism and facilitating the breakdown and elimination of visceral fat [154]. Systematic reviews of randomized controlled trials in overweight and obese populations have concluded that high-dose probiotic supplementation represents a promising intervention, with moderate but significant reductions in BMI as the most commonly reported outcome [155,156,157,158].

The role of specific beneficial gut microbial species in the development and management of obesity has garnered considerable scientific interest [159]. *Bifidobacterium* and *Lactobacillus* are among the most commonly studied probiotics in both animal models and obese human subjects due to their notable antibiotic resistance and low pathogenic potential [155,160,161]. Probiotics within the *Lactobacillaceae* family have demonstrated significant efficacy in reducing adipose tissue mass while enhancing lipid metabolism, primarily via the stimulation of fatty acid oxidation and inhibition of LPL activity [162]. Sanchis-Chordà et al. [163] investigated the effects of the probiotic *Bifidobacterium pseudocatenulatum* on cardiometabolic risk factors, inflammatory cytokines, and GM composition in obese children with insulin resistance, reporting a significant reduction in BMI post-intervention. Furthermore, probiotic administration has been shown to modulate GM composition, particularly affecting the abundance of *Rikenellaceae* family members, with a notable predominance of the genus *Alistipes*.

In a randomized trial involving overweight or obese insulin-resistant individuals, administration of pasteurized *A. muciniphila* for three months resulted in a modest reduction in BW alongside a significant improvement in insulin sensitivity [164]. Previously, *A. muciniphila* abundance was linked to fasting glucose levels, visceral and subcutaneous adiposity, and weight management in a 6-week calorie-restriction intervention involving 49 overweight and obese participants [165]. Conversely, a reduction in *F. prausnitzii* has been associated with a decreased capacity to counteract obesity-related inflammatory processes [166]. This species contributes to the restoration of intestinal barrier integrity and is currently being explored for its therapeutic potential [167]. Similarly, *Alistipes* and *Roseburia* genera have shown promise as therapeutic targets in the treatment of obesity and metabolic disorders [168].

Contrary to the generally health-promoting, anti-obesogenic effects attributed to probiotics, some studies have reported negligible or even obesogenic outcomes associated with their use [169,170]. These conflicting results may stem from variations in probiotic strains, as well as differences in host factors such as age and baseline BW. Consequently, there is a clear need for more rigorous and comprehensive randomized controlled trials to definitively determine the efficacy of probiotics in obesity management across diverse populations.

Synbiotics, defined as the combination of prebiotics and probiotics, have been shown to exert synergistic effects that exceed those of their components alone [171,172]. Their primary advantage lies in enhancing probiotic survival and viability within the gastrointestinal tract. In addition, synbiotics play a pivotal role in modulating gut metabolic activity by promoting microbiota growth, maintaining intestinal integrity, and inhibiting pathogenic species [161]. Moreover, they also increase levels of carbon disulfides, ketones, methyl acetates, and SCFAs, which may confer health benefits to the host [173].

The therapeutic potential of synbiotics in managing obesity, T2D, and associated metabolic disorders has garnered considerable attention in recent research [174]. Notably, Rajkumar et al. [175] demonstrated that co-supplementation with omega-3 fatty acids and a high-dose probiotic mixture comprising *Bifidobacterium*, *Lactobacillus*, and *Streptococcus* species led to significant improvements in plasma lipid concentrations, GM composition, insulin sensitivity, and inflammatory markers in overweight subjects. While probiotic supplementation alone induced favorable microbial shifts, omega-3 fatty acid administration without probiotics did not produce comparable effects. Despite these promising findings, current evidence regarding synbiotic interventions remains limited, and variables such as the timing of administration appear to critically influence therapeutic outcomes. Table 2 presents a summary of recent clinical studies investigating the effects of probiotics and synbiotics on obesity.

### 6.2. Fecal Microbiota Transplantation

Standard FMT in humans entails the transfer of intestinal microbiota from a donor to a recipient via various delivery methods, including oral fecal capsules, colonoscopy, nasogastric or nasojejunal tubes, enemas, sigmoidoscopy, or rectal tubes [192]. Successful FMT is characterized by the sustained establishment of a donor-like microbiome in the recipient for at least 3 months post-transplantation [193].

This procedure has demonstrated transferable behavioral phenotypes and established links between GM composition and metabolic disorders [194], with emerging evidence suggesting potential applications in obesity management [195]. The exploration of FMT for obesity and its related comorbidities was driven by the ability of GM interventions to modulate glucose metabolism, enhance SCFA production, and reduce systemic inflammation [196]. Notably, Kootte et al. [197] reported that FMT administration significantly improved insulin responsiveness in individuals with metabolic syndrome. Similarly, Vrieze et al. [198] observed a marked increase in insulin sensitivity 6 weeks post-FMT compared to baseline levels in metabolic syndrome patients.

**Figure 3:**
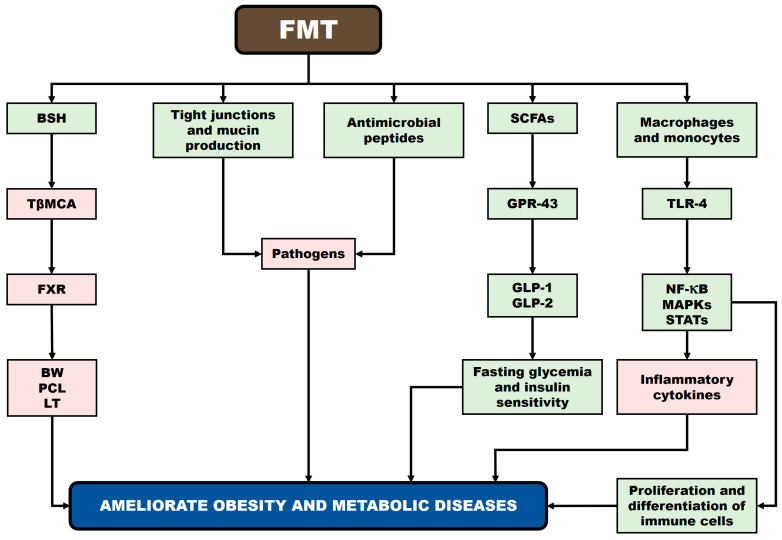
Potential mechanisms underlying the relationship between FMT and obesity. Rectangles in green: increase. Rectangles in red: decrease. BSH: bile salt hydrolase; TβMCA: tauro-β-muricholic acid; FXR: farnesoid X receptor; BW: body weight; PCL: plasma cholesterol; LTs: liver triglycerides; SCFAs: short-chain fatty acids; GPR43: G-protein-coupled receptor 43; GLP-1 and GLP-2: glucagon-like peptides 1 and 2; TLR4: Toll-like receptor 4; NF-κB: nuclear factor-kappa B; MAPK: mitogen-activated protein kinase; and STAT: signal transducer and activator of transcription.

Kang and Cai [199] proposed several mechanisms underlying the effects of FMT on obesity: (i) FMT alters GM composition, influencing bacterial bile salt hydrolase (BSH) activity, which decreases levels of tauro-β-muricholic acid (TβMCA), which constitutes an antagonist of the FXR, thereby reducing BW gain, plasma cholesterol, and LTs; (ii) FMT may suppress pathogen growth by stimulating antimicrobial peptide secretion and strengthening the intestinal barrier through maintenance of tight junctions and increased mucin production; (iii) SCFAs produced post-FMT activate GPR-43, promoting secretion of GLP-1 and GLP-2, which regulate fasting glycemia and insulin sensitivity; and (iv) FMT may modulate immune responses via TLR-4 on macrophages and monocytes, triggering NF-κB, mitogen-activated protein kinase (MAPK), and signal transducer and activator of transcription (STAT) pathways, thereby influencing inflammatory cytokine secretion and immune cell proliferation and differentiation. Figure 3 presents potential mechanisms underlying the relationship between FMT and obesity (according to Kang and Cai [199]).

Regarding obesity interventions, the Gut Bugs Trial was conducted to assess the efficacy of FMT in improving metabolic function and treating obesity in 87 children and adolescents affected by this condition [200]. The primary outcome was the change in BMI six weeks post-FMT, administered via orally encapsulated fecal microbiota from same-sex donors, with a follow-up period of 26 weeks. While the intervention significantly reduced abdominal adiposity, no improvements were observed in insulin sensitivity, lipid profiles, BMI, liver function, blood pressure, inflammatory markers, or overall gut health. Mild adverse effects occurred, predominantly loose stools in 10% of participants, but no serious adverse events were reported. Complementing these findings, Zecheng et al. [201] demonstrated through a meta-analysis and systematic review that FMT improves blood glucose metabolism, insulin resistance, blood pressure, cholesterol levels, SCFA production, and inflammatory status in overweight individuals.

Allegretti et al. [202] conducted a double-blind trial involving 22 obese patients without diabetes, nonalcoholic steatohepatitis, or metabolic syndrome. Participants received oral FMT capsules (30 capsules at week 4 and week 8) derived from a single lean donor, with follow-up extending to week 26. The treatment was well tolerated and resulted in sustained alterations in GM composition and BA profiles resembling those of the donor. However, no significant differences in mean BMI were observed between groups at week 12. Similarly, Yu et al. [203] performed a 12-week double-blind, randomized, placebo-controlled pilot trial at a US academic center, enrolling 24 obese adults with mild-to-moderate insulin resistance who received FMT capsules from a healthy lean donor for six weeks. Although FMT led to GM engraftment in most recipients lasting at least 12 weeks, no clinically meaningful metabolic improvements were detected during the study period.

In turn, Hartstra et al. [204] demonstrated that FMT from Roux-en-Y gastric bypass donors modulate the microbiota–gut–brain axis in individuals with obesity. Recipients exhibited alterations in dopamine and serotonin transporter levels alongside shifts in GM composition, highlighting the potential of FMT to target the microbiota–gut–brain axis as a therapeutic approach for obesity [205]. Despite its ability to improve metabolic parameters, FMT has not been consistently associated with weight reduction in obese patients [206]. In addition, autologous FMT, where patients receive their own “healthy” microbiota collected prior to surgery during recovery, has emerged as a promising strategy to restore individualized GM composition [207].

Despite its currently limited clinical application, optimized FMT interventions remain a key focus of ongoing research. One promising strategy involves pretreatment with broad-spectrum antibiotic cocktails, which reduce the recipient’s native gut microbiota, thereby creating a less competitive environment and enhancing the engraftment and efficacy of transplanted microbes [207]. However, the specific effects of FMT on the microbiota–gut–brain axis components require further elucidation. Recently, Borrego-Ruiz and Borrego [208] outlined several critical questions for future investigation. First, the underlying mechanisms of FMT’s therapeutic efficacy remain unclear, partly due to the complex composition of FMT material, which includes viable and non-viable bacteria, as well as other microorganisms comprising the virome and mycobiome. Second, while FMT contains numerous metabolites (e.g., Bas and SCFAs) and proteins, the precise components responsible for its clinical effects are yet to be identified. Third, advances in FMT technology have introduced novel methods such as Washed Microbiota Transplantation (WMT) and spore transplantation. In this respect, controlled comparative studies and long-term safety evaluations of these approaches are still lacking.

## 7. Current Therapeutic Tools for the Treatment of Obesity

### 7.1. Pharmacological Therapy

Pharmacological therapy for obesity is intended to support weight reduction and improve metabolic health when lifestyle modifications alone are insufficient. Current approved pharmacotherapies are reported to induce a 5–15% reduction in BW. Anti-obesity and glucose-lowering agents primarily act by promoting satiety (e.g., liraglutide, lorcaserin, pramlintide, setmelanotide, and sibutramine) or by leading to nutrient malabsorption (e.g., orlistat) [209].

Once pharmacotherapy has been initiated and the recommended or maximum tolerated dose is achieved, treatment efficacy should be reassessed after 3–4 months [210]. This evaluation should consider whether weight-loss goals have been met, as well as improvements in metabolic parameters, obesity-related comorbidities, and quality of life. Although currently there are no pharmacological treatment guidelines specifically designed for older adults with obesity, commonly used agents in clinical settings include pancreatic lipase inhibitors, GLP-1 receptor agonists, dopamine/norepinephrine reuptake inhibitors, and opioid receptor antagonists [210,211]. Given the increasing availability of anti-obesity medications, the therapeutic landscape is becoming progressively complex. Consequently, the selection of a specific pharmacological agent should not rely solely on efficacy, but must also consider patient preferences, comorbidities, tolerability, and potential drug–disease or drug–drug interactions [212].

Various studies have highlighted a potential therapeutic role for antibiotics, particularly vancomycin, in addressing obesity-related microbial dysbiosis by reducing TNF-α levels in murine models and enhancing insulin sensitivity in humans [213,214]. Beyond antibiotics, numerous non-antimicrobial pharmaceuticals, including hormones, antidepressants, and antihistamines, have been shown to modulate GM composition. Indeed, a broad survey identified 44 drug categories that significantly influence the GM, among them metformin, statins, and laxatives [215]. While current anti-obesity medications exert marked effects on GM composition, their specific interactions with the gut–brain axis remain insufficiently explored.

A range of unimolecular GLP-1R/GcgR dual agonists have been developed using the glucagon amino acid sequence as a basis. These glucagon-based chimeric peptides were generated through targeted amino acid substitutions designed to enhance potency at the GLP-1R and to achieve balanced co-agonism at both receptors. The therapeutic potential of GLP-1R/GcgR co-agonism in obesity management has been underscored by studies evaluating its effects in combination with leptin therapy [209]. GLP-1 receptor agonists have demonstrated substantial benefits in improving glycemic control, promoting weight loss, and mitigating metabolic complications. Emerging therapeutic strategies, including dual and triple incretin receptor agonists, are showing superior efficacy in the management of both diabetes and obesity. Nevertheless, important challenges remain, such as the need to optimize treatment outcomes, consider inter-individual variability, and enhance long-term adherence [216,217]. Notably, several studies have reported that this novel therapeutic approach exhibits lower efficacy than both retatrutide and tirzepatide in reducing BW and WC in individuals with obesity or overweight [218].

### 7.2. Bariatric Surgery

Bariatric surgery is widely acknowledged as a highly effective intervention for obesity and its associated comorbidities, particularly in cases where other conventional therapies have failed. Emerging evidence indicates that its metabolic benefits are, in part, mediated by alterations in GM composition. Post-surgical shifts in the GM include increased production of SCFAs, enhanced abundance of *Akkermansia* and members of the family *Streptococcaceae*, and a concomitant reduction in bacterial genera belonging to the family *Bacteroidaceae*. Regarding dietary changes, patients exhibit lower absolute carbohydrate intake in both the short and long term [219].

In addition to its immediate metabolic benefits, bariatric surgery exerts long-term effects on the GM, which may contribute to sustained weight loss and metabolic improvements [6,220,221,222]. These enduring effects are thought to result from multiple physiological changes post-surgery, including reduced gastric volume, increased luminal pH, and diminished efficiency in dietary energy extraction. Typically, these modifications lead to a reduction in Bacillota, a phylum whose high abundance is associated with obesity, and also to an increase in Pseudomonadota, which are linked to improvements in systemic inflammation, glucose homeostasis, and weight reduction. Moreover, there is often an observed rise in *Enterobacteriaceae*, a family negatively correlated with cholesterol levels and positively associated with weight loss [45,223,224,225].

A longitudinal study examining the fecal microbiome of obese patients before and after bariatric surgery revealed a post-operative increase in the abundance of the genera *Butyricimonas*, *Parabacteroides*, and *Slackia*. Conversely, genera such as *Acinetobacter*, *Coprococcus*, *Lachnospira*, *Lactococcus*, *Megamonas*, *Oribacterium*, and *Phascolarctobacterium*, which were predominant in non-surgical obese individuals, showed a marked decline following the intervention [220]. These microbial shifts are likely attributable to the post-surgical rise in intestinal pH and oxygen levels, which inhibit anaerobic bacteria and promote the proliferation of aerobic taxa such as Pseudomonadota [226]. Notably, patients who experienced greater weight loss and long-term weight maintenance exhibited a higher diversity in core microbiota. These individuals had increased relative abundance of genera such as *Alkaliphilus*, *Butyrivibrio*, *Cetobacterium*, *Lachnospira*, *Pseudoalteromonas*, and *Sarcina* [182].

### 7.3. Physical Activity

Physical activity is widely acknowledged as a fundamental component in the prevention and management of overweight and obesity in both children and adults, as it produces weight loss, improves fitness, reduces cardiometabolic risk, and enhances quality of life [227,228]. School-based interventions have been widely explored as platforms for promoting physical activity and healthy habits in children and adolescents. While short-term school interventions tend to improve knowledge regarding nutrition and physical activity, their behavioral impact remains modest unless supported by broader community involvement [229,230]. Multi-component strategies that integrate physical education, dietary counseling, and health promotion across school and community settings have shown more promising results, particularly among adolescents from disadvantaged backgrounds, improving adiposity markers and physical fitness without necessarily reducing BMI [231,232]. Notably, programs that incorporate circuit training, high-intensity interval training, or innovative approaches such as gamified platforms have demonstrated improvements in cardiometabolic risk factors, body composition, and muscular strength in children and adolescents with moderate to severe obesity [233,234,235,236]. Ultimately, for school-based interventions to effectively prevent obesity and foster lifelong physical activity habits, they must be longitudinal, centered on direct physical engagement, and designed to develop not only physical competence but also the motivation and knowledge that underpin physical literacy [237]. In adults with overweight or obesity, physical activity plays a key role in improving health beyond weight loss, contributing significantly to cardiovascular fitness, metabolic function, mental well-being, and reductions in all-cause mortality [238,239,240]. Both aerobic and resistance training, especially when combined, have been shown to improve cardiorespiratory fitness and muscle strength, with high-intensity interval training and aerobic-based programs offering the most notable cardiovascular benefits [241]. These effects are observed even without major changes in weight [240]. Interventions that use adaptive goals, mobile health technologies, and reward-based systems have proven effective in increasing physical activity and overcoming common barriers such as low motivation or self-efficacy [242,243,244].

Research has shown that physical activity can modulate the GM, resulting in improved metabolic and immune function, as evidenced in both animal models and human studies [245]. Distinct microbial compositions have been observed between individuals with normal weight and those with obesity. Post-exercise interventions reveal significant shifts in GM composition. In this respect, *Bacteroides* were found to be more dominant in obese individuals, whereas *Faecalibacterium* and *Lachnospira* were more prevalent among normal-weight individuals. Moreover, exercise-induced modifications in GM composition were shown to revert after the cessation of physical activity, suggesting that the microbiota is dynamically responsive to lifestyle interventions [246]. Similarly, Motiani et al. [247] conducted a study involving 27 sedentary individuals with obesity and reported that moderate-to-high-intensity physical activity resulted in a decreased B/B ratio. This shift was accompanied by an increased abundance of *Bacteroides* and a concomitant reduction in *Blautia* and *Clostridium* abundance.

### 7.4. Behavioral Therapies

Standard behavioral therapies for obesity include motivational interviewing and cognitive–behavioral therapy, both grounded in principles such as goal setting, self-monitoring, and stimulus control [248]. While motivational interviewing has been proposed as a useful tool in obesity management, its effectiveness remains uncertain, particularly given the lack of evidence supporting its impact on weight outcomes when added to behavioral weight management programs [249]. In addition, the resource-intensive nature of motivational interviewing, requiring substantial staff training and increased intervention time, limits its practicality in routine clinical settings [249]. In turn, cognitive–behavioral therapy has demonstrated moderate efficacy in promoting lifestyle change and weight reduction when compared to usual-care controls, suggesting its relative value in obesity interventions [250]. In this context, integrating components that address psychological well-being and health-related quality of life may enhance treatment outcomes, highlighting the importance of a more holistic approach to obesity care [251].

The GM may play a potential role in influencing the success of behavioral therapies. Through the microbiota–gut–brain axis, microbe-derived metabolites affecting neural pathways can influence appetite regulation, mood, and stress responses [252], factors that directly impact behavioral adherence and weight management. Therefore, integrating GM modulation strategies with behavioral therapies could enhance treatment efficacy for obesity by addressing certain psychological and physiological determinants.

## 8. Emerging Therapeutic Approaches for Obesity

### 8.1. Brown Adipocyte Thermogenesis

A promising therapeutic strategy for addressing obesity and its associated metabolic disorders is the stimulation of brown adipocyte thermogenesis. This mechanism enhances energy expenditure by promoting the browning of white adipose tissue, a process mediated in part by increased production of microbial metabolites such as acetate and lactate [253]. These metabolites have been shown to activate thermogenic pathways, thereby facilitating fat oxidation and energy dissipation. Nevertheless, a major challenge in advancing this approach lies in the identification and development of pharmacological agents capable of reliably inducing thermogenesis in humans, which holds the potential to markedly improve obesity treatment outcomes [254]. Moreover, critical questions remain regarding whether enhanced brown adipose tissue activity serves as a driver or a consequence of weight loss and the potential risks associated with sympathetic nervous system-mediated non-shivering thermogenesis [255].

### 8.2. Precision Nutrition

In recent years, precision nutrition has emerged as a promising strategy to adapt dietary recommendations based on individual factors such as genetics, epigenetics, the GM, and lifestyle. This personalized approach has demonstrated utility in managing metabolic disorders and obesity [256]. Central to this strategy is the identification of biological markers that are particular to each individual, allowing the design of more customized and effective nutritional interventions. Such targeted approaches are expected to improve the efficacy of both prevention and treatment. The current literature shows that weight-loss interventions yield better outcomes when genetic profiles are incorporated into the treatment plan [257]. For instance, patients with a history of unsuccessful weight loss who underwent nutrigenetic testing for 24 variants across 19 metabolism-related genes achieved significantly greater long-term weight loss and improved fasting glucose levels compared to non-tested controls [258].

A growing body of evidence highlights that imbalances in GM composition directly impact lipid metabolism, immune and inflammatory pathways, and appetite regulation [10,122]. Carvalho et al. [26] emphasized the interaction between the GM and diet, illustrating how dietary patterns such as Mediterranean and vegetarian fiber-rich diets enhance SCFA production and promote the growth of beneficial bacteria linked to weight regulation and reduced inflammation. Current data indicate that the abundance of taxa such as *A. muciniphila* and *F. prausnitzii* correlates strongly with metabolic health and responsiveness to weight-loss interventions [45]. As reported in the literature, diet remains the primary modulator of GM composition [95]. However, other factors such as genetic and epigenetic traits, body composition, maternal nutritional status, mode of delivery, physical activity, and drug consumption also play significant roles in shaping the GM [52]. Despite challenges such as cost and variability in individual response, the integration of omics technologies, artificial intelligence, and machine learning holds great promise for identifying specific microbial and genetic profiles, thereby enabling the development of personalized nutritional interventions optimized to meet individual metabolic needs.

### 8.3. Vagus Nerve Stimulation

Decreased vagus nerve activity has been implicated in the development of hemodynamic and metabolic dysfunction associated with obesity [27]. In high-fat diet-induced obesity, vagal regulation of hepatic glucose production is impaired, while pharmacological activation of cholinergic signaling pathways or direct vagus nerve stimulation suppresses appetite and promotes weight loss [259,260].

Clinical studies using implanted nerve stimulators have demonstrated that vagus nerve stimulation can induce significant weight reduction in obese patients. In this respect, Huerta et al. [27] applied peripheral focused ultrasound stimulation to the liver of C57BL/6J mice fed a high-fat, high-carbohydrate Western diet. Daily ultrasound treatment over eight weeks resulted in significant body weight loss and reductions in circulating lipid levels. In addition, the intervention also alleviated adipokine dysregulation. Moreover, hepatic ultrasound stimulation markedly decreased hepatic cytokine expression and leukocyte infiltration. These findings reveal the potential of hepatic-focused ultrasound as a novel, noninvasive therapeutic approach for obesity management.

## 9. Discussion

This review presents an updated synthesis of current evidence on the role of the GM in human obesity, including diet, prebiotics, probiotics, synbiotics, and FMT as therapeutic strategies for GM modulation. To our knowledge, no previous review has addressed the GM in human obesity in such a comprehensive and integrative manner, covering microbial alterations, underlying mechanisms, dietary influences, prebiotic modulation, and microbiome-targeted therapies, as well as current and emerging therapeutic approaches. While microbial-based interventions are emerging as promising tools for managing obesity and related pathologies, several challenges must be addressed to enhance their efficacy and reproducibility. One critical issue is the heterogeneity in patient responses, as individuals often exhibit varying outcomes despite receiving similar treatments, a discrepancy influenced by factors such as baseline microbiota composition and host-specific variables. Genetic differences among individuals may further modulate immune responses, nutrient metabolism, and host–microbiota interactions, thereby impacting therapeutic effectiveness. Moreover, a significant limitation lies in the insufficient mechanistic understanding of these interventions. For instance, the FMT interventions reviewed were generally performed in pilot trials with small sample sizes and heterogeneous study populations. In turn, clinically significant outcomes, such as weight loss and alterations in BMI, are typically assessed up to 12 months following an intervention [202,203]. However, the efficacy of a single donor in terms of inducing a sustained effect remains uncertain. In contrast, multiple FMTs did not produce substantial metabolic improvements in subjects with metabolic syndrome [204]. As noted, metabolic and microbiome responses among FMT recipients exhibited considerable variability, and the studies lacked sufficient statistical power to analyze outcomes by recipient subgroups or to evaluate donor-specific effects. Finally, the FMT protocols did not include dietary interventions, and the majority of participants consumed typical high-fat, low-fiber Western diets. Therefore, the influence of diet needs to be validated in larger randomized controlled trials encompassing participants from diverse ethnic backgrounds [261].

Although the beneficial effects of microbial therapies are increasingly being recognized, the precise molecular pathways, particularly those involving microbial metabolites such as SCFAs and BAs, and their interactions with host metabolic networks remain insufficiently elucidated. Integrating multi-omics technologies, including metagenomics, metabolomics, and proteomics, may offer comprehensive insights into the dynamic interplay between the GM, the immune system, and host metabolism. Ultimately, the development of an integrative conceptual framework that incorporates microbial metabolites, immune modulation, and systemic metabolic regulation is essential for optimizing microbiota-targeted interventions aimed at improving metabolic health.

It is increasingly evident that inter-individual variability in GM composition significantly influences responses to dietary interventions aimed at obesity management. Studies assessing fecal microbiota before and after dietary interventions have revealed the stability of the microbiome over time and demonstrated marked heterogeneity in microbial and physiological responses among individuals subjected to identical diets [262]. This variability means that two individuals adhering to the same diet may experience markedly different physiological and microbial outcomes. For instance, Hjorth et al. [263] demonstrated that individuals with *Prevotella*-dominant microbiomes exhibited greater BF loss on a Nordic diet compared to those with *Bacteroides*-dominant microbiota, underscoring the role of microbial profiles in modulating dietary responsiveness. These findings underscore the potential of precision nutrition approaches that customize dietary strategies based on individual microbiome characteristics to maximize metabolic benefits. Thus, dietary interventions for obesity should not overlook inter-individual heterogeneity in their design.

Precision nutrition aims to customize dietary interventions by predicting individual metabolic responses, typically involving three main strategies: (i) increasing fiber intake, (ii) restricting caloric intake, and (ii) supplementing with prebiotics and probiotics [264]. Although these resemble conventional approaches, precision nutrition differs in that it incorporates assessment of prior individual responses, such as shifts in GM composition or glycemic control, to design customized plans, as opposed to a standardized dietary model. The success of this approach is often evaluated through microbial markers (e.g., increases in *Prevotella* abundance), anthropometric outcomes (e.g., weight loss, BMI, and FM reduction), and metabolic indicators like glycemic responses [265]. For instance, Zeevi et al. [266] developed an algorithm that accurately predicted postprandial glucose responses based on individual microbiome and clinical data, demonstrating superior predictive capacity compared to traditional metrics such as the glycemic index. Despite these advances, the application of personalized nutrition within the context of the microbiota–gut–brain axis remains underexplored. There is a critical need for long-term studies to investigate how sustained dietary modifications affect the GM and associated biochemical pathways, particularly those influencing hunger hormones, inflammatory cytokines, and other neuroendocrine signals implicated in obesity and T2D.

Obesity has been linked to dysregulated glycolysis, impaired oxidative phosphorylation, increased production of reactive oxygen species, and mitochondrial fragmentation. Current therapeutic approaches aim to reduce cellular stress and restore metabolic homeostasis, primarily through antioxidants or by modulating mitochondrial dynamics. Considering this, mitochondria-targeted therapies hold promise as a novel strategy to correct metabolic dysfunction in affected individuals [267]. However, to date, interventions specifically targeting mitochondrial dynamics for obesity treatment have yielded limited success, highlighting a gap and an opportunity for further research in this area. Notably, one study demonstrated that a synthetic sphingolipid could modulate mitochondrial dynamics in mice fed a high-fat diet, leading to restoration of healthy body weight [268]. This finding suggests that physiological manipulation of mitochondrial function may represent a viable therapeutic option. Nevertheless, the complexity of mitochondrial involvement in obesity-related metabolic disturbances requires comprehensive investigation to elucidate precise mechanisms and optimize targeted treatments. Future research should focus on understanding the interplay between mitochondrial dynamics, energy metabolism, and systemic metabolic regulation to fully unlock the therapeutic potential of mitochondria-targeted interventions in obesity.

Within the framework of this discussion, it is essential to acknowledge a key interconnection between obesity, mental health, and the GM, which serves as a central regulator of both metabolic and neuropsychological functions. Diet, as a major external factor, shapes the composition and metabolic activity of the GM, thereby influencing host health through modulation of immune development, nutrient metabolism, and bioactive molecule synthesis [93]. The GM is implicated in the etiology of various mental disorders, such as anxiety and depression, with substantial evidence linking its composition and functional dynamics to nervous system development and regulation [252]. Communication between the central nervous system and the GM occurs via immune, endocrine, and neural routes, including the vagal nerve, forming a complex gut–brain axis that impacts metabolic homeostasis and mental well-being [252]. Research indicates that obesity is associated with reductions in quality of life and increases in adverse mental health outcomes, underscoring a predominant influence of obesity on psychological distress [269,270,271]. Epidemiological data further indicate that individuals with obesity or overweight exhibit significantly diminished quality of life and heightened depression scores compared to normal-weight counterparts, with obesity exerting the most pronounced effect [269]. The high co-prevalence of obesity and mental illness poses a critical public health challenge, positioning the GM as a promising target for interventions aimed at improving metabolic and psychological outcomes. In recent years, the emerging field of nutritional psychiatry has begun to reshape clinical approaches, emphasizing the necessity for scientifically rigorous evaluation of dietary supplements and nutraceuticals due to their widespread use among individuals with and without mental health disorders [272]. Psychobiotics, a novel class of psychotropics comprising live microorganisms and bioactive substances, have demonstrated efficacy in ameliorating psychological conditions such as anxiety, stress, and depression, as well as in improving cognitive function, sleep quality, emotional regulation, and mood symptoms [273,274]. Given that diet constitutes a key element in obesity management, precision nutrition offers the opportunity not only to improve obesity-related metabolic dysfunctions but also to enhance mental health outcomes by modulating the GM. Concurrently, microbiome-based therapies, which have shown promise in managing obesity, could be strategically directed to address comorbid psychological conditions, particularly depression, which is highly prevalent among individuals with obesity. In this respect, a strictly vegetarian ketogenic diet has been proposed as a potential nutritional intervention to promote overall health, including improvements in both metabolic and mental health outcomes [108]. Considering the potential efficacy of vegetarian and ketogenic diets in obesity management, the implementation of a strictly vegetarian ketogenic dietary protocol, complemented by structured physical exercise and appropriately designed microbiome-based therapies, could constitute a promising, non-pharmacological, and non-surgical strategy for the effective treatment of obesity. This integrative approach may synergistically modulate metabolic regulation and the gut–brain axis, addressing the complex etiology of obesity and its associated mental health comorbidities. Furthermore, integrating components that address individual motivation, environmental factors, and long-term behavioral reinforcement may be key to improving maintenance outcomes.

Beyond its well-established medical implications, obesity often exposes individuals to stigma and discrimination across various social domains, including the workplace, education, and interpersonal relationships. Indeed, this pervasive stigma has become widely normalized and frequently goes unrecognized as a legitimate form of discrimination [275]. Addressing obesity stigma requires a coordinated societal response, beginning with dispelling common misconceptions about its causes and the rejection of simplistic notions of personal responsibility. A shift toward greater societal understanding and empathy may foster environments in which stigmatizing behaviors are challenged and ultimately eradicated [275]. In the healthcare setting, actionable strategies to mitigate stigma include shifting the clinical focus away from weight, adopting patient-centered communication, ensuring inclusive facilities and equipment, and improving access to specialized obesity care by removing administrative barriers [276]. In Spain, recent data reveal that individuals with obesity often report more aversion toward the condition than those of normal weight, and younger populations report higher frequencies of stigmatizing encounters [277]. These findings underscore the urgent need to confront negative societal beliefs and attitudes as an integral component of public health efforts. It is also noteworthy that even after bariatric surgery, patients continue to experience high levels of stigma, highlighting the persistence of weight-related prejudice despite clinical improvements [277]. While public visibility and advocacy from individuals living with obesity are essential to drive systemic changes and resource allocation, caution is warranted when individuals with self-induced overweight attempt to affiliate with this group. Although excessive weight due to unhealthy lifestyle choices remains a concern, equating it with medically defined obesity threatens the legitimacy of the condition and may overload limited healthcare resources intended for those truly affected. Sustainable progress will depend not only on individual and healthcare-level interventions but also on comprehensive public education campaigns and policy reforms that promote inclusivity and dismantle structural barriers contributing to obesity-related stigma. Ultimately, scientific progress driven by ongoing research must provide future approaches that effectively address obesity and mitigate its consequences, aiming to reduce its impact sufficiently to ensure an improved quality of life across affected demographics.

Lastly, it should be noted that obesity in adolescents is strongly associated with an increased likelihood of experiencing bullying compared to their healthy-weight peers [278]. The co-occurrence of obesity and bullying among children and adolescents remains a critical global concern, with evidence indicating a close relationship between these factors during key developmental stages [279]. While multiple strategies have been developed to manage and treat obesity, including cognitive and behavioral interventions, it seems that the role of certain emotional processes in the etiology and maintenance of this condition is not sufficiently considered. Bullying can provoke intense emotional responses such as humiliation, shame, and anger [280], which may contribute to maladaptive coping mechanisms, including emotional eating, which is defined as the tendency to consume food in response to negative emotions [281]. Since this phenomenon can exacerbate weight gain and complicate obesity management, integrating an assessment of the emotional experiences and coping strategies of individuals with obesity, particularly in the context of bullying and psychosocial stressors, could provide critical insights into underlying psychological mechanisms. Therefore, this approach would foster the development of more effective, comprehensive interventions addressing both the physiological and emotional dimensions of obesity.

Despite the comprehensive coverage of current findings, this review presents several limitations: (i) the heterogeneity in intervention types, including differences in intensity, duration, and methodology, as well as variability in outcome measures; (ii) sample sizes varied substantially, ranging from small pilot studies to large cohort trials; (iii) the uneven geographic distribution of the included studies, with limited representation from developing regions; (iv) inconsistencies in adverse event reporting and inadequate follow-up in several studies; (v) difficulties in translating microbiota-targeted therapies from controlled trials into routine clinical practice, including the lack of standardized operating procedures across diverse patient populations, variability in the quality and reproducibility of biological specimens, and infrastructural constraints, especially regarding advanced therapies such as FMT; (vi) limited clinical accessibility due to specialized equipment requirements for microbiota intervention preparation, storage, and delivery; (vii) burdens on healthcare providers and systems arising from the need for customized approaches in personalized microbiome-based therapies; (viii) high costs associated with microbiome analysis, therapeutic formulation, and delivery, particularly in resource-constrained settings.

Building on these identified limitations, it is also necessary to acknowledge that the clinical translation of emerging therapies faces several critical challenges, including the development of standardized treatment protocols, the assurance of patient safety, and the confirmation of long-term efficacy. These challenges must be considered in conjunction with future research priorities, such as clarifying inter-individual variability, elucidating the still opaque mechanisms of obesity and related diseases, and integrating multi-omics data to capture complex host–microbiome interactions. Furthermore, the precise mechanisms through which microbiome-targeted interventions exert clinical effects remain incompletely understood, limiting mechanistic insight and rational optimization. A central barrier to the development of standardized treatment processes lies in the heterogeneity of research findings, which highlights the need for consistent, evidence-based clinical protocols. The absence of standardized outcome measures further complicates comparisons across studies and the synthesis of results. Addressing this issue requires the establishment of clear clinical guidelines, robust quality control frameworks, and active regulatory oversight to ensure the reproducibility and reliability of new therapeutic and diagnostic approaches. Equally important is the identification and mitigation of adverse events before widespread adoption. This necessitates an exhaustive safety assessment pipeline, incorporating preclinical toxicology studies and rigorous post-market surveillance systems, to ensure that therapies are safe for long-term use. In addition to safety, a major challenge is to demonstrate sustained therapeutic efficacy. Short-term improvements must be substantiated by evidence of durable benefits, which can only be achieved through long-term follow-up studies tracking patient outcomes, disease recurrence, and the persistence of therapeutic effects. This is particularly important in chronic, multifactorial conditions such as obesity, where treatment success depends on stability over time. In this respect, many studies have relatively short intervention periods, which restricts evaluation of long-term efficacy and safety. Emerging innovations will gradually improve the scalability and reproducibility of microbiome-based therapies. Successful implementation may demonstrate their feasibility and clinical utility. Efforts should prioritize the standardization of methodologies, reductions in costs through technological innovation, and the generation of high-quality, large-scale clinical evidence to support widespread application.

## 10. Conclusions

The composition of the GM is altered in obesity and characterized by reduced microbial diversity and inconsistent shifts in dominant bacterial phyla, which collectively contribute to metabolic dysregulation. The GM contributes to obesity pathogenesis through multiple mechanisms, including SCFA-mediated regulation of energy homeostasis and insulin sensitivity, LPS-induced chronic inflammation, modulation of host metabolic and appetite-regulating genes, and alterations in BA signaling via FXR. In addition, GM-driven inhibition of FIAF promotes LPL activity and fat storage in adipose tissue.

Dietary patterns exert a profound influence on GM composition and function, with plant-based diets enhancing SCFA production and conferring protective effects against obesity and its comorbidities. Prebiotics modulate GM composition by promoting beneficial bacteria, such as *Bifidobacterium* and *A*. *muciniphila*, which are linked to improved metabolic outcomes, reduced inflammation, and enhanced insulin sensitivity. In addition, microbiome-based therapeutics, including probiotics, synbiotics, and FMT, have demonstrated potential in modulating key metabolic and inflammatory pathways associated with obesity. However, further research is needed to fully elucidate their mechanisms and optimize clinical applications.

As the scientific understanding of the human GM continues to advance, the integration of microbiome-based therapies into standard clinical practice is poised to become increasingly feasible and therapeutically transformative, particularly for obesity, a complex condition that demands innovative and customized interventions.

## Figures and Tables

**Table 1:** Effects of prebiotics on animals and humans with obesity.

Study	Prebiotic Treatment	Outcomes
Preclinical		
Bai et al. [131]	Oligosaccharides from the plant *Codonopsis pilosula* for 16 weeks to HFD-fed mice	The prebiotic treatment decreased fat accumulation and BW and improved glucose tolerance in HFD-fed obese mice. Increased the proportion of the beneficial bacteria *Muribaculaceae*, *Alistipes*, and *Clostridium* and decreased the abundance of the harmful bacteria *Rikenella*, *Enterobacteriaceae*, *Collinsella*, and *Megasphaera*.
Huang et al. [132]	Fermented Tartary buckwheat dietary fiber for 14 weeks to HFD-fed-mice	Decreased microbial imbalance and increased microbial diversity. Increase in members of the taxa Verrucomicrobiota, Clostridiales, and *Muribaculaceae*, as well genus *Lactobacillus*.
Li et al. [133]	Crude guava polysaccharides for 11 weeks to HFD-fed mice	Alleviated BW gain, visceral obesity, insulin resistance, and meta-inflammation and reduced levels of serum cholesterol and triglycerides. Increased the abundance of beneficial bacteria such as *Clostridium XlVa*, *Parvibacter*, and *Enterorhabdus* and reduced the proportion of inflammation-related bacteria *Mucispirillum.* Increased SCFA synthesis, particularly butyrate.
Ma et al. [134]	Polysaccharides from the oyster *Crassostrea gigas* for 10 weeks to HFD-fed obese mice	Polysaccharides reduced weight gain, dyslipidemia, and metabolic endotoxemia in HFD-fed obese mice and enhanced the production of SCFAs. Increased beneficial bacteria, such as *Bifidobacterium*, *Lactobacillus*, *Dobosiella*, and *Faecalibaculum* and decreased harmful bacteria, including *Erysipelatoclostridium*, *Helicobacter*, and *Mucispirillum*.
Mo et al. [135]	Insoluble yeast β glucans for 24 weeks to obese rats	Prebiotic ameliorated weight gain, systemic inflammation, dyslipidemia, insulin resistance, and glucose intolerance. Restored HFD-induced gut dysbiosis and changed levels of LPS and SCFAs in the HFD-fed rats.
Oh et al. [136]	Neoagarooligosaccharides for 12 weeks to obese Sprague-Dawley rats	Reduced BW gain and metabolic syndrome associated with obesity. Increased the abundances of some taxa negatively associated with obesity, particularly *Eubacterium fissicatena* and *Ruminococcaceae UCG-005.101*.
Wei et al. [137]	Polysaccharides from the seaweed *Enteromorpha clathrate* for 4 weeks to HFD-fed mice	Prebiotics improved intestinal dysbiosis and reshaped the structure of the GM in obese mice. Increased the abundance of the butyrate-producing bacterium *Eubacterium xylanophilum*.
Clinical		
Canfora et al. [138]	Galacto-oligosaccharides for 12 weeks to overweight/obese, prediabetic individuals (*n* = 44)	Increased the abundance of *Bifidobacterium*, but no effect on microbial diversity or richness of GM.
Edrisi et al. [139]	Rice bran/rice husk powder for 12 weeks to overweight and obese adults (*n* = 105)	Decreased weight, BMI, and WC.
Lambert et al. [140]	Yellow pea fiber for 12 weeks to overweight overweight/obese adults (*n* = 50)	Reduced BF and energy intake. Increased insulin levels. No effect on GM.
Machado et al. [141]	Fructo-oligosaccharides for 6 weeks to overweight adults (*n* = 26)	Decreased BW, WC, BF, and waist-to-height ratio. Improved bowel function.
Neyrinck et al. [142]	Inulin-type fructans for 12 weeks to obese patients (*n* = 24)	Decreased a fecal marker of intestinal inflammation. Increased *Bifidobacterium*.
Nicolucci et al. [143]	Oligofructose-enriched inulin for 16 weeks to overweight/obese children (aged 7–12 years) (*n* = 42)	Decreased BW, BF, percent trunk fat, percent BF, serum triglycerides, and interleukin 6. Reduced the abundance of *Bacteroides vulgatus* and increased *Bifidobacterium* spp.
Pol et al. [144]	Oligofructose for 12 weeks to overweight/obese adults (*n* = 55)	Reduced appetite. No effect on other parameters related to obesity, including energy intake and BW.
Reimer et al. [145]	Inulin-type fructans for 12 weeks to overweight and obese adults (*n* = 125)	Increased *Bifidobacterium* spp. and satiety.
Vaghef- Mehrabany et al. [146]	Inulin for 8 weeks to obese women with MDD (*n* = 45)	Reduced BW, BMI, WC, hip circumference, and FM in the experimental group.
van de Beek et al. [147]	Inulin for 2 days to obese men (*n* = 14)	Increased fat oxidation and SCFAs. Decreased plasma free fatty acids, plasma glucose, and insulin. No effects on plasma triglycerides, GLP-1, PYY, or hunger and satiety scores.

HFD: high-fat diet; GM: gut microbiome; BMI: body mass index; BW: body weight; WC: waist circumference; BF: body fat; FM: fat mass; MDD: major depressive disorder; SCFAs: short-chain fatty acids; GLP-1: glucagon-like peptide-1; PYY: peptide YY.

**Table 2:** Effects of probiotics and synbiotics in humans with obesity.

Study	Treatment	Outcomes
Probiotics		
Banach et al. [176]	Probiotic: yogurt containing *Lactobacillus acidophilus* strain LA-5 and *Bifidobacterium animalis* subsp. *lactis* (formerly *B. lactis*) strain BB-12. *n* = 54 healthy overweight and obese adults, 12 weeks.	Reduced BW and FM.
Kim et al. [177]	Probiotic: *Lactobacillus paragasseri* (formerly *L. gasseri*). *n* = 90 healthy overweight and obese adults, 12 weeks.	Reduced FM and WC.
Minami et al. [178]	Probiotic: *Bifidobacterium breve* strain B3. *n* = 18 pre-obese individuals, 12 weeks.	The intake of probiotic slightly decreased triglyceride levels and improved HDL cholesterol from the baseline.
Narmaki et al. [179]	Probiotic cocktail: *L. acidophilus*, *Lacticaseibacillus rhamnosus*, *Limosilactobacillus reuteri*, *B. animalis* subsp. *lactis*, *B. bifidum*, and *B. longum.* *n* = 62 obese females, 6 + 6 weeks.	Reduced the scores for BF, BMI, BW, TF, WC, and WHR.
Razmpoosh et al. [180]	Probiotics: pasteurized kashk *L. acidophilus* strain LA-5 and *B. animalis* subsp. *lactis* strain BB-12. *n* = 65 obese women, 8 weeks.	Reduced BFM, BFP, BMI, BW, and WC.
Sanchis-Chordà et al., [163]	Probiotic: *Bifidobacterium pseudocatenulatum* strain CECT 7765. *n * = 48 obese children, 13 weeks.	Probiotic intake improved inflammatory status and insulin resistance. These effects were parallel to increases in bacterial groups associated with a lean phenotype.
Zarrati et al. [181]	Probiotic: yogurt containing *L. acidophilus* strain LA-5, *Lacticaseibacillus casei* strain DN001, and *B. animalis* subsp. *lactis* strain BB-12. *n* = 56 obese individuals, 8 weeks.	Reduced BFP.
Synbiotics		
Angelino et al. [182]	Probiotic: *Bacillus coagulans* strain GBI-30. Prebiotic: β glucans. *n* = 41 sedentary overweight and obese individuals, 12 weeks.	Reduced plasma GGT, plasma LDL/HDL cholesterol ratio, and *Bifidobacterium* spp. Increased *F. prausnitzii* abundance.
Gutiérrez-Repiso et al. [183]	Probiotics: *B. animalis* subsp. *lactis*, *B. longum*, and *L. rhamnosus*. Prebiotic: unspecified plant fiber. *n* = 33 obese patients, 8 + 8 weeks.	Reduced BW.
Janczy et al. [184]	Probiotics: *B. animalis* subsp. *lactis*, *L. acidophilus*, *Lactobacillus salivarius*, *Lactobacillus lactis* subsp. *lactis*, *rhamnosus*, *Lacticaseibacillus paracasei*, and *Lactiplantibacillus plantarum*. Prebiotic: FOS + inulin. *n* = 56 obese individuals, 12 weeks.	Reduced BMI, BW, and FM.
Kanazawa et al. [185]	Probiotic: *Lacticaseibacillus paracasei* strain Shirota and *Bifidobacterium breve* strain Yakult. Prebiotic: GOS. *n* = 88 obese individuals with T2D, 24 weeks.	No significant changes in inflammatory markers. However, synbiotic administration improved the gut environment in obese patients with T2D.
Kassaian et al. [186]	Probiotics: *B. animalis* subsp. *lactis*, *B. bifidum*, *B. longum*, and *L. acidophilus.* Prebiotic: inulin. *n* = 120 prediabetic individuals, 24 weeks.	Reduced fasting insulin levels, fasting plasma glucose, glycated hemoglobin, and insulin resistance. Increased QUICKI.
Krumbeck et al. [187]	Probiotics: *B. animalis* subsp. *lactis* strain BB-12 and *B. adolescentis* strain IVS-1. Prebiotic: GOS. *n* = 114 overweight individuals, 3 weeks.	Although the synbiotic did not demonstrate functional synergism, the findings clearly showed that the pro- and prebiotic components by themselves improved markers of colonic permeability.
Mohammadi-Sartang et al. [188]	Probiotic: yogurt containing *B. animalis* subsp. *lactis* strain BB-12. Prebiotic: inulin + whey + calcium + vitamin D3. *n* = 90 obese individuals with metabolic syndrome, 10 weeks.	Reduced FM.
Perraudeau et al. [189]	Probiotics: *Akkermansia muciniphila*, *Clostridium beijerinckii*, *Clostridium butyricum*, *Bifidobacterium infantis*, and *Anaerobutyricum hallii* Prebiotic: inulin. *n* = 76 patients with T2D, 12 weeks.	Improved the glucose total area under the curve and glycated hemoglobin.
Sergeev et al. [190]	Probiotics: *B. animalis* subsp. *lactis* strain UABIa-12, *B. bifidum* strain UABb-10, *B. longum* strain UABIa-14, and *L. acidophilus* strain DDS-I. Prebiotic: GOS. *n* = 20 obese adults, 12 weeks.	Non-significant differences between intervention and control groups in BFP, BMI, BW, and FM.
Xavier-Santos et al. [191]	Probiotic: *L. acidophilus* strain LA-5. Prebiotic: inulin + FOS. *n* = 45 adults with metabolic syndrome, 8 weeks.	The daily intake of mousse (control) and synbiotic led to significant reductions in total cholesterol and HDL-cholesterol, as well as immunoglobulins (A and M) and interleukin-1 beta, in both groups.

BF: body fat; BFM: body fat mass; BFP: body fat percentage; BMI: body mass index; BW: body weight; FM: fat mass; FOS: fructo-oligosaccharides; GGT: γ glutamyltransferase; GOS: galacto-oligosaccharides; HDL: high-density lipoprotein; LDL: low-density lipoprotein; QUICKI: quantitative insulin sensitivity check index; TF: trunk fat; T2D; type 2 diabetes; WC: waist circumference; WHR: weight-to-height ratio.

## Abbreviations

The following abbreviations are used in this manuscript:

GM	Gut microbiome
T2D	Type 2 diabetes mellitus
FMT	Fecal microbiota transplantation
B/B	Bacillota/Bacteroidota
BMI	Body mass index
BF	Body fat
WC	Waist circumference
VFA	Visceral fatty area
SCFAs	Short-chain fatty acids
GPRs	G-protein-coupled receptors
PPARs	Peroxisome proliferator-activated receptors
GLP-1	Glucagon-like peptide-1
GLP-2	Glucagon-like peptide-2
PYY	Peptide YY
AMPK	AMP-activated protein kinase pathway
FIAF	Fasting-induced adipose factor
LPL	Lipoprotein lipase
LPS	Lipopolysaccharide
LBP	LPS-binding protein
TLR4	Toll-like receptor 4
NF-κB	Nuclear factor-κ B
TNF-α	Tumor necrosis factor-α
*LYPLAL1*	Lysophospholipase-like 1 gene
BAs	Bile acids
LTs	Liver triglycerides
FXR	Farnesoid X receptor
BW	Body weight
FM	Fat mass
LDL	Low-density lipoprotein
HOMA-IR	Homeostasis model assessment
HFD	High-fat diet
MDD	Major depressive disorder
BSH	Bile salt hydrolase
TβMCA	Tauro-β-muricholic acid
MAPK	Mitogen-activated protein kinase
STAT	Signal transducer and activator of transcription
WMT	Washed Microbiota Transplantation
